# *Hemiphyllodactylus ziegleri* sp. nov. (Squamata, Gekkonidae), a new karst-dwelling gecko species from Son La Province, Vietnam

**DOI:** 10.3897/zookeys.1268.174678

**Published:** 2026-02-04

**Authors:** Anh Van Pham, Truong Quang Nguyen, Cuong The Pham, Hanh Thi Ngo, Minh Duc Le

**Affiliations:** 1 Faculty of Environmental Sciences, University of Science, Vietnam National University, Hanoi, 334 Nguyen Trai Road, Hanoi, Vietnam University of Science, Vietnam National University Hanoi Vietnam; 2 Institute of Biology, Vietnam Academy of Science and Technology, 18 Hoang Quoc Viet Road, Hanoi 10072, Vietnam Vietnam National University Hanoi Vietnam; 3 Graduate University of Science and Technology, Vietnam Academy of Science and Technology, 18 Hoang Quoc Viet Road, Hanoi 11416, Vietnam Vietnam Academy of Science and Technology Hanoi Vietnam; 4 Central Institute for Natural Resources and Environmental Studies, Vietnam National University, Hanoi, 19 Le Thanh Tong, Hanoi 11021, Vietnam American Museum of Natural History New York United States of America; 5 Department of Herpetology, American Museum of Natural History, Central Park West at 79th Street, New York 10024 USA Graduate University of Science and Technology Hanoi Vietnam

**Keywords:** Copia Nature Reserve, morphology, ND2 mitochondrial gene, northern Vietnam, taxonomy

## Abstract

Based on morphological and molecular analyses, a new species of *Hemiphyllodactylus* is described from Son La Province, northern Vietnam. *Hemiphyllodactylus ziegleri***sp. nov**. differs from its sister taxon, a paraphyletic *H. yunnanensis*, and other members of the genus *Hemiphyllodactylus* by at least 14% in terms of genetic divergence based on a fragment of the ND2 mitochondrial gene and a combination of the following morphological characters: SVL of adult males 38.7–41.9 mm and adult females 47.0–49.3 mm; dorsal scale rows 21–27; ventral scale rows 12–16; chin scales bordering mental and first infralabial distinctly enlarged; digital lamellae formula 3444 (forefoot) and 4555/4 (hindfoot); femoral pores absent; precloacal pores 21–23 in males, absent in females; cloacal spur single, present in both sexes; dorsal trunk pattern yellowish grey; body with a discontinuous light dorsolateral stripe. Our discovery increases the number of species within the genus to 73.

## Introduction

Son La Province is covered by 599,000 hectares of natural forest (The People’s Committee of Son La Province 2019). Recent biodiversity research in Son La Province showed that the evergreen forest in the province harbors a high level of reptile diversity. During the past decade, seven new species of reptiles have been described from Son La Province, namely *Achalinus quangi* Pham, Pham, Le, Ngo, Ong, Ziegler & Nguyen; *A. timi* Ziegler, Nguyen, Pham, Nguyen, Pham, Van Schingen, Nguyen & Le; *A. vanhoensis* Ha, Ziegler, Sy, Le, Nguyen & Luu; *Cyrtodactylus sonlaensis* Nguyen, Pham, Ziegler, Ngo & Le; *C. taybacensis* Pham, Le, Ngo, Ziegler & Nguyen; *Hemiphyllodactylus vanhoensis* Luu, Hoang, Ha, Grismer, Murdoch, Sitthivong, Phimpasone & Grismer; *H. yenchauensis* Luu, Sitthivong, Hoang, Ha, Ha, Xayasith, Ziegler, Grismer; and *Scincella truongi* Pham, Ziegler, Pham, Hoang, Ngo & Le (Nguyen et al. 2017; Pham et al. 2019, 2023, 2025; Ziegler et al. 2019; Ha et al. 2022; Luu et al. 2024, 2025).

The genus *Hemiphyllodactylus* Bleeker, 1860 currently comprises 72 recognized species, with a distribution range from southern India and Sri Lanka, through Indochina and Southeast Asia, to the western Pacific region (Luu et al. 2024; Uetz et al. 2025). Because of its cryptic lifestyle and small body size, *Hemiphyllodactylus* is one of the most poorly known groups of geckos with 61 species (or 85% of the total extant species) described during the last ten years. In Vietnam, 11 species of the genus *Hemiphyllodactylus* are currently recognized, namely *H. yunnanensis* Boulenger; *H. zugi* Nguyen, Lehmann, Le, Duong, Bonkowski & Ziegler; *H. banaensis* Ngo, Grismer, Thai & Wood; *H. bonkowskii* and *H. ngocsonensis* Nguyen, Do, Ngo, Pham, Pham, Le & Ziegler; *H. nahangensis* Do, Pham, Phan, Le, Ziegler & Nguyen; *H. dalatensis* Do, Nguyen, Le, Pham, Ziegler & Nguyen; *H. lungcuensis* Luu, Nguyen, Do, Pham, Hoang, Nguyen, Le, Ziegler, Grismer & Grismer; *H. vanhoensis* Luu, Hoang, Ha, Grismer, Murdoch, Sitthivong, Phimpasone & Grismer; *H. cattien* Yushchenko, Grismer, Bragin, Dac & Poyarkov; and *H. yenchauensis* Luu, Sitthivong, Hoang, Ha, Ha, Xayasith, Ziegler & Grismer (Do et al. 2020, 2021; Nguyen et al. 2020; Luu et at al. 2023, 2024, 2025; Uetz et al. 2025).

Our recent field work in karst forests of Son La Province, northern Vietnam led to the discovery of an unnamed population of *Hemiphyllodactylus* from Copia Nature Reserve (NR). Based on morphological and molecular phylogenetic data, we herein describe it as a new species from Vietnam.

## Materials and methods

### Sampling

Field surveys were conducted in Copia NR, Son La Province, northern Vietnam, in June 2013 and September 2014. Geckos were anaesthetized and euthanized in a closed vessel with a piece of cotton wool containing ethyl acetate (Simmons 2002), fixed in 85% ethanol and subsequently stored in 70% ethanol. Specimens were subsequently deposited in the collections of the University of Science (**HUS**), Vietnam National University, Hanoi (**VNU**), Vietnam and Institute of Biology (**IB**), Hanoi, Vietnam.

### Molecular data and phylogenetic analyses

We sequenced four new samples from Copia NR, Son La Province. Sequences from 29 related species were included as ingroup taxa and *H. harterti* was chosen as the outgroup (Luu et al. 2025).

Tissue samples were extracted using the Dneasy blood and tissue kit, Qiagen (Hilden, Germany). Extracted DNA was amplified by DreamTaq PCR mastermix (Thermo Fisher Scientific, Lithuania). A fragment of the mitochondrial gene ND2 (NADH dehydrogenase subunit 2) was amplified using the primer pair ND2f101A 5’-CAACAGAAGCCACAACAAAAT-3’ and HemiR 5’-GAAGAAGAGGCTTGGKAGGCT-3’ (Nguyen et al. 2013). The newly generated sequences were deposited in GenBank (Suppl. material 1).

The PCR volume consisted of 21 µl (10 µl of mastermix, 5 µl of water, 2 µl of each primer at 10 pmol/µl, and 2 µl of DNA). PCR condition was: 95 °C for 5 min to active the taq; with 35 cycles at 95 °C for 30s, 48 °C for 45s, 72 °C for 60s; and the final extension at 72 °C for 6 min. PCR products were subjected to electrophoresis through a 1% agarose gel. Gels were stained for 10 min in 1× TBE buffer at 2 pg/ml of ethidium-bromide and visualized under UV light. Successful amplifications were purified to eliminate PCR components using GeneJET PCR Purification Kit (Thermo Fisher Scientific, Lithuania). Purified PCR products were sent to 1^st^ BASE (Selangor, Malaysia) for sequencing.

Sequences generated in this study were aligned using De Novo Assemble function in the program Geneious v. 7.1.8 (Kearse et al. 2012). After sequences were aligned by Clustal X v. 2 (Thompson et al. 1997), data were analyzed using maximum parsimony (MP) as implemented in PAUP*4.0b10 (Swofford 2001), maximum likelihood (ML) in IQ-TREE v. 3.0.1 (Wong et al. 2025), and Bayesian inference (BI) in MrBayes v. 3.2.7 (Ronquist et al. 2012), respectively. Settings for these analyses followed Le et al. (2006), except that the number of generations in the Bayesian analysis was increased to 1 × 10^7^ and the cut-off point for the burn-in function was set to 25% of the total number of trees generated in the Bayesian analysis. The optimal model for nucleotide evolution was set to GTR+G+I as selected by jModelTest v. 2.1.10 (Darriba et al. 2012) and used for BI analyses. Nodal support was evaluated using bootstrap replication (BP) as calculated in PAUP (1,000 replications and 100 random taxon addition), ultrafast Bootstrap (UFB) (1,000 replications) in IQ-TREE, and posterior probability (PP) in MrBayes v. 3.2.7. Uncorrected pairwise divergences were calculated in PAUP*4.0b10. Nodal support was evaluated using BP as estimated in PAUP, UFB in IQ–TREE v. 3.0.1, and PP in MrBayes v. 3.2.7. UFB > 95, and PP ≥ 0.95, and BP ≥ 70% were regarded as strong support for a clade (Hillis and Bull 1993; Ronquist et al. 2012; Hoang et al. 2018).

### Morphological characters

Terminology of morphological characters followed Zug (2010) and Grismer et al. (2013, 2025). Measurements were taken with a digital caliper to the nearest 0.1 mm. The following abbreviations were used:

**SVL** snout-vent length (from the tip of snout to the vent);

**TaL** tail length (from the vent to the tip of the tail, original or regenerated);

**TrunkL** trunk length (from the posterior margin of the forelimb at its insertion point on the body to the anterior margin of the hindlimb at its insertion point on the body);

**HeadL** head length (from the posterior margin of the retroarticular process of the lower jaw to the tip of the snout);

**HeadW** head width (measured at the angle of the jaws);

**EyeD** eye diameter (the greatest horizontal diameter of the eyeball);

**SnEye** snout-eye length (from anteriormost margin of the eyeball to the tip of the snout);

**NarEye** naris-eye length (from the anterior margin of the eyeball to the posterior margin of the external naris);

**SnW** internarial width (measured between the nares across the rostral and mental scales, respectively);

**EarD** ear diameter (maximum diameter of ear);

**Ventral scales** the number of longitudinal ventral scales at midbody contained within one eye diameter;

**Dorsal scales** the number of longitudinal dorsal scales at midbody contained within one eye diameter;

**The number of subdigital lamellae** on the first finger and first toe;

**Lamellae formula**, determined as the number of U–shaped subdigital lamellae on the digital pads on digits 2–5 of the hands and feet;

**The total number of precloacal and femoral pores** (the contiguous rows of femoral and precloacal scales bearing pores); and

The number of cloacal spurs.

Bilateral scale counts are given as left/right.

### Statistical analyses

For the statistical analyses, the newly discovered population was compared to their closest relatives based on the phylogeny of *Hemiphyllodactylus*. Raw morphological data used for the analyses were obtained from the specimens collected in Copia NR and from 25 specimens representing the five other *Hemiphyllodactylus* species, available from previous studies (Luu et al. 2024, 2025). These raw data are presented in Suppl. material 2.

All statistical analyses were conducted in R v. 4.4.2 (R Core Team 2024). To remove the effects of allometry in the morphometric characters, morphometric data were also normalized to adjust raw data of morphometrics using the following equation: X_adj_ = log(X) – ß[log(SVL)-log(SVL_mean_)], where X_adj_ = adjusted value; X = measured value; ß = unstandardized regression coefficient for each population and SVL_mean_ = overall average SVL of two populations (Thorpe 1975, 1983; Turan 1999; Lleonart et al. 2000; Chan and Grismer 2022; Luu et al. 2025). A multiple factor analysis (MFA; Grismer 2025) was conducted to assess morphospace between individuals from Copia NR and their close relatives using the “FactorMineR” package (Husson et al. 2017) and visualized with “Factoextra” (Kassambara and Mundt 2017). A non-parametric permutation multivariate analysis of variance (PERMANOVA) from “PairwiseAdonis” package in R (available at https://github.com/pmartinezarbizu/pairwiseAdonis/tree/master/pairwiseAdonis) was used to determine if the centroid locations and group clusters of each species were statistically different from each other based on the MFA load scores of dimensions 1–5 (Anderson 2017; Oksanen et al. 2020). An Euclidean (dis) similarity matrix was calculated using 50,000 permutations (Grismer et al. 2024). A pairwise post hoc test was also applied to estimate the differences between the studied species pairs. A p-value of < 0.05 was considered to indicate a significant difference between the studied taxa.

## Results

### Phylogenetic analyses

**Figure 1.**
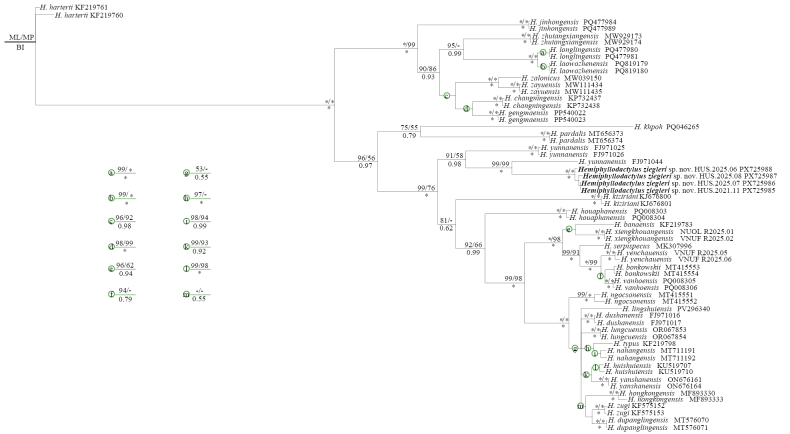
Phylogram based on the Bayesian topology. Number above and below branches are MP/ML bootstrap values and Bayesian posterior probabilities, respectively. Asterisk and dash represent 100% and <50% values, respectively. Branches without numbers receive perfect statistical support from all analyses.

The final matrix consists of 618 aligned characters, of which 368 characters were parsimony informative; 227 characters were constant; and 23 variable characters were parsimony uninformative. MP analysis of the dataset recovered 26 most parsimonious trees with 1598 steps (CI = 0.41; RI = 0.79). In the ML analysis, the log likelihood of the best tree found was -7755.56. Tree topologies derived from three analyses were very similar, and samples from the new population were recovered as a sister taxon to a paraphyletic *Hemiphyllodactylus yunnanensis* from Yunnan Province, China, with high statistical support values from all analyses (UFB = 99; BP = 99; PP = 1.0). The two taxa formed a lineage closely related to another clade of *H. yunnanensis* from Yunnan, China, with weak support from MP and ML (UFB = 91; BP = 58; PP = 0.98) (Fig. 1). Our phylogenies also resemble those of Luu et al. (2024) and Luu et al. (2025), except for the position of *H. houaphanensis*. Sequences of new samples were at least 14% divergent from those of other species within the genus (Suppl. material 3).

### Statistical analyses

**Figure 2.**
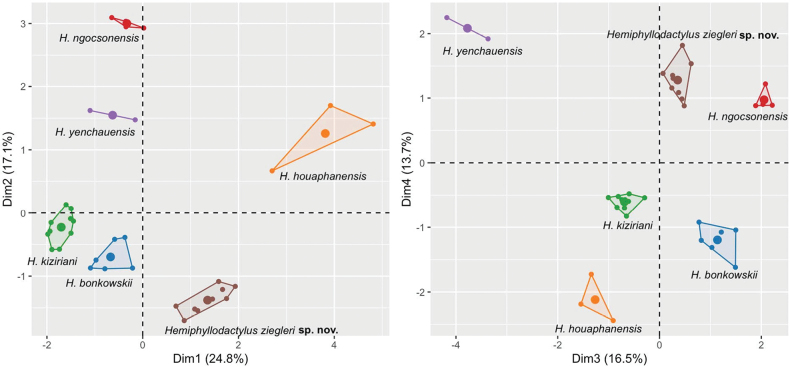
MFA scatter plots showing the morphospatial relationships among selected the *Hemiphyllodactylus* species along the first four dimensions.

In the MFA analysis, the four dimensions explain 24.8%, 17.1%, 16.5%, and 13.7% of the total variation, respectively, accounting for 72.1% of the total variation in the dataset (Fig. 2). The MFA analysis revealed that, although the Copia NR population overlaps with *H. ngocsonensis* in the fourth dimension, they are separated from each other in the first three dimensions, which account for 58.4% of the total variation (Fig. 2). Additionally, the Copia NR population is also separated from all other closely related species of *Hemiphyllodactylus*, except for *H. yunnanensis* due to the lack of available morphological data, along the ordination of the first four dimensions. The PERMANOVA analysis also indicated that the new population in Copia NR differs significantly in morphospace from closely related species of *Hemiphyllodactylus* (Table 1).

**Table 1.** Summary statistics from the PERMANOVA analysis from the loadings of the MFA comparing *Hemiphyllodactylus ziegleri* sp. nov. to closely related species.

Species pairs	F.Model	R2	p.value	p.adjusted
*Hemiphyllodactylus ziegleri* sp. nov. – *H. bonkowskii*	161.28	0.93	0.00018	0.002
*Hemiphyllodactylus ziegleri* sp. nov. – *H. houaphanensis*	96.62	0.91	0.0047	0.04
*Hemiphyllodactylus ziegleri* sp. nov. – *H. kiziriani*	75.05	0.94	0.00002	0.0003
*Hemiphyllodactylus ziegleri* sp. nov. – *H. ngocsonensis*	69.26	0.95	0.0013	0.015
*Hemiphyllodactylus ziegleri* sp. nov. – *H. yenchauensis*	61.65	0.94	0.018	0.096

### Morphological comparisons

We compared the undescribed gecko species from Son La Province, Vietnam with other members of the genus *Hemiphyllodactylus* based on examination of specimens (see Suppl. material 2) and data obtained from the literature (Boulenger 1903; Barbour 1924; Smith 1935; Taylor 1963; Zhou et al. 1981; Bourret 2009; Zug 2010; Grismer et al. 2013, 2014, 2015, 2018, 2020, 2021, 2024, 2025; Nguyen et al. 2013, 2014, 2020; Guo et al. 2015; Cobos et al. 2016; Sukprasert et al. 2018; Sung et al. 2018; Eliades et al. 2019; Do et al. 2020, 2021; Agung et al. 2021, 2022; Luu et al. 2023, 2024, 2025; Wang et al. 2025).

### Taxonomic account

#### Hemiphyllodactylus ziegleri sp. nov.

Taxon classificationAnimaliaSquamataGekkonidae

3945F607-99BD-5882-A58A-9753B1374A72

https://zoobank.org/946C7128-AEFD-4BEC-AA03-22757836BAEC

Figs 3, 5

##### Type material.

***Holotype***. HUS 2025. 06 (Field number PAT 188) (Fig. 4), adult male, collected on 15 July 2013 by A.V. Pham, on a limestone cliff in the corn field (20°43.480'N, 104°54.344'E, at an elevation of 1020 m a.s.l.), Nong Vai Village, Co Ma Commune in Copia NR, Son La Province, northern Vietnam. ***Paratypes***. HUS. 2025.07 (Field number PAT 95), IB R.6431 (Field PAT.186), HUS.2025.08 (Field PAT.187), HUS.2025.09 (Field number PAT. 92), HUS.2025.10 (Field number PAT. 93), IB R.6432 (Field number PAT. 94) (Fig. 5A), and HUS.2025.11 (Field PAT.220) (Fig. 5B), adult female, the same data as the holotype; IB R.6433 (Field SL.2014.33), adult male, collected on 16 September 2014 by T. Q. Nguyen and A.V. Pham on a limestone cliff, Nong Vai Village, Co Ma Commune in Copia NR (20°43.481'N, 104°54.349'E, at an elevation of 1020 m a.s.l.).

**Figure 3.**
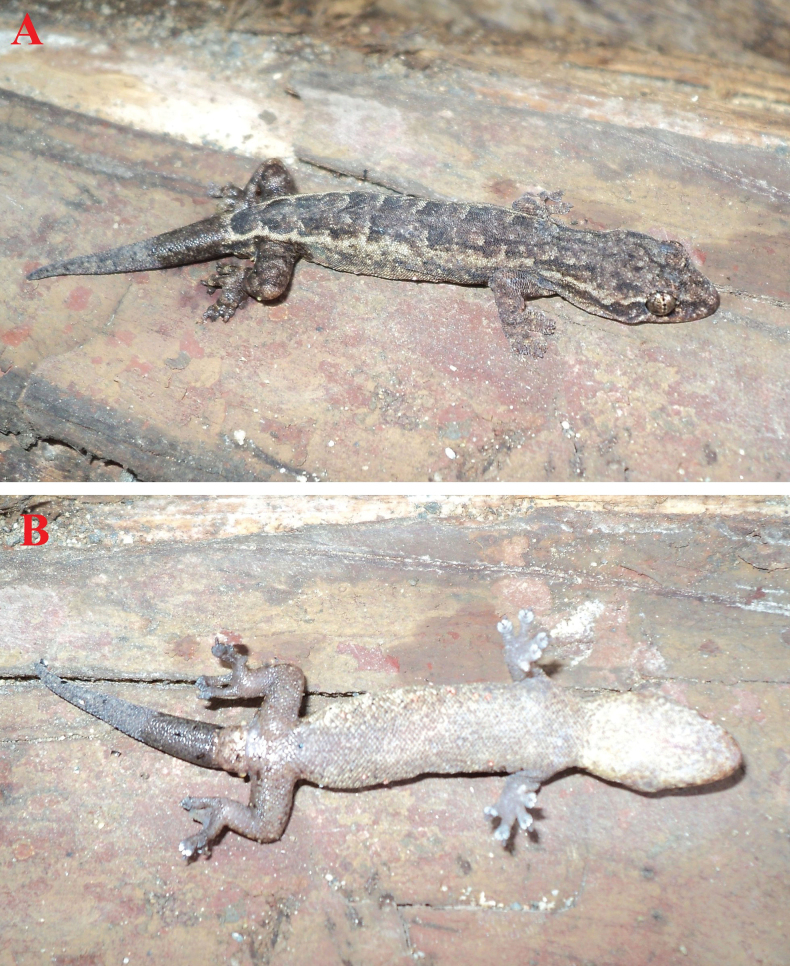
The male holotype of *Hemiphyllodactylus ziegleri* sp. nov. (HUS.2025.06) in life. **A**. Dorsal view; **B**. Ventral view. Photos: A.V.P.

**Figure 4.**
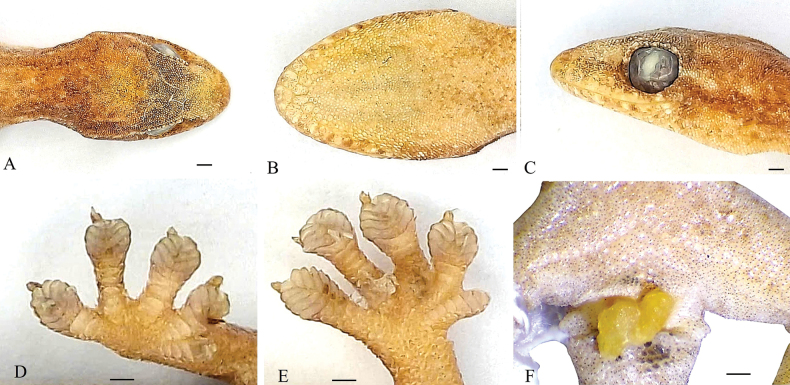
The male holotype of *Hemiphyllodactylus ziegleri* sp. nov. (HUS.2025.06) in preservative: Head. **A**. Dorsal view; **B**. Ventral view; **C**. Lateral view; **D**. Subdigital view of right hand; **E**. Subdigital view of right foot; **F**. Precloacal region with precloacal and femoral pores in a continuous series. Photos: A.V.P. Scale bar: 1 mm.

**Figure 5.**
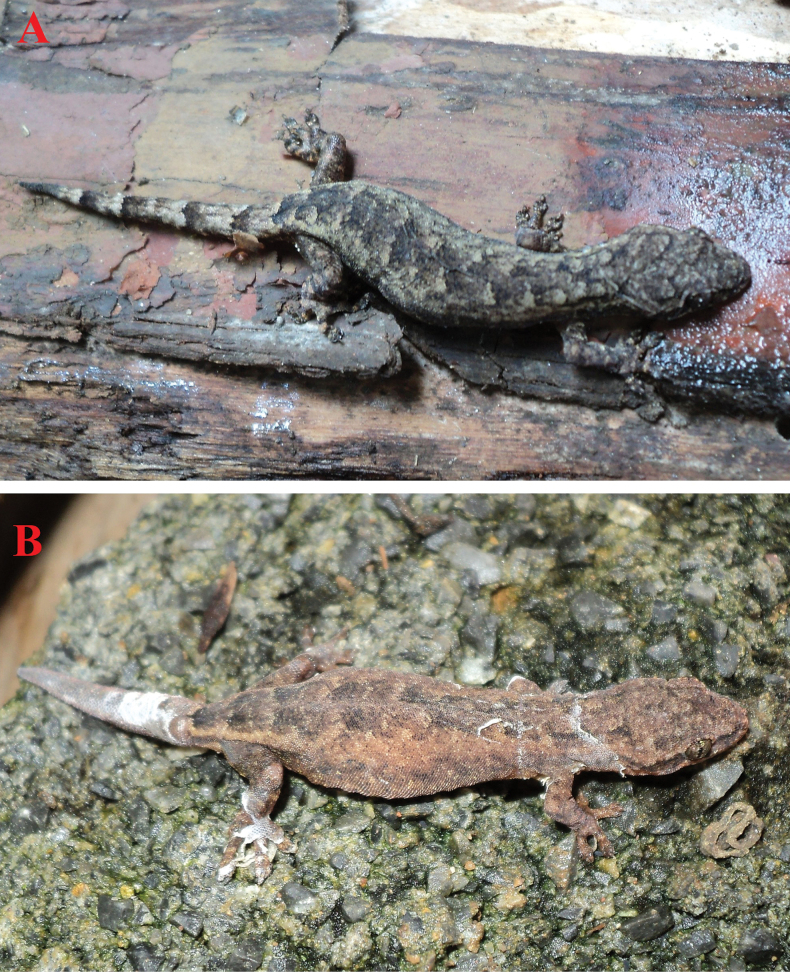
*Hemiphyllodactylus ziegleri* sp. nov. in life. **A**. The male paratype (IB R.6432); **B**. The female paratype (IB R.6433). Photos: A.V.P.

##### Diagnosis.

A bisexual taxon; SVL of adult males 38.7–41.9 mm, adult females 47.0–49.3 mm; dorsal scale rows 21–27; ventral scale rows 12–16; chin scales bordering mental and first infralabial distinctly enlarged; digital lamellae formula 3444 (forefoot) and 4555/4 (hindfoot);femoral and precloacal pores 21–23 in males, absent in females; cloacal spur single, present in both sexes; dorsal trunk pattern yellowish grey; body with a discontinuous light dorsolateral stripe.

##### Description of holotype.

***Body*** dorsolaterally flattened, size small SVL 41.9 mm, tail regenerated (TaL 16.2 mm+), trunk length (TrunkL 20.08 mm), head longer than wide (HeadL 9.62 mm, HeadW 7.24 mm), eye moderate (EyeD 2.68 mm), ear opening oblique (EarD 0.91 mm), nar-eye length (NarEye 3.24 mm), snout-eye length (SnEye 4.51 mm), internarial distance (SnW 1.59 mm). ***Proportions***: TrunkL/SVL 0.48, HeadL/SVL 0.23, HeadW/SVL 0.17, HeadW/HeadL 0.75, SnEye/HeadL 0.47, NarEye/HeadL 0.34, EyeD/HeadL 0.28, SnW/HeadL 0.17, EyeD/NarEye 0.83, SnW/HeadW 0.22; pupil vertical; ear opening oblique, oval, approximately 37% of the eye diameter, without bordering enlarged scales.

***Scalation***. Rostral very large, wider than high (RW 1.62 mm, RH 0.95 mm), with a shallow suture; supralabials 10/10, enlarged from rostral to below eye, smaller in subocular rictus; naris in contact with rostral, first supralabial, supranasal, and three nasals posteriorly on each side; supranasals separated from each other by two small granular internasals; snout flat, covered by granular scales; infralabials 9/9; mental triangular, wider than long, bordered laterally by first infralabials and posteriorly by two large postmentals; bordered laterally by first infralabials and posteriorly by two large postmentals; two large postmentals in contact with mental and first infralabials anteriorly; seven chin scales; gular scales small, subimbricate; throat and pectoral scales which grade into slightly larger, subimbricate. Dorsal scales small, granular, 25–27 scale rows at midbody contained within one eye diameter, enlarged tubercles absent; ventrolateral folds absent; ventral scales, flat, larger than dorsal scales; enlarged, femoral and precloacal scales; 21 pore-bearing femoral and precloacal scales, in a continuous row; cloacal spur one; dorsal surface of fore and hindlimbs covered with granular scales; terminal two phalanges free, claws absent on first finger and on first toe, present on second to fifth digit of fore and hindfoot; pads of digits II–V each with large triangular lamellae, digital formula 3443 (forefoot) and 4544 (hindfoot); lamellae five on first fingers, five on first toes; dorsal caudal scales granular; subcaudals flat, slightly larger than dorsal caudal scales.

##### Coloration in preservative.

Dorsal surface of head and body yellowish grey; a light brown streak originating from posterior corner of eye to the neck; neck and dorsum with a row of light spots on each side; dorsal surface of limbs grey with dark bars; upper lips with dark bars; lower lips white; throat white; venter and precloacal region cream with small dark brown dots; testes white, unpigmented. For color and pattern in life see Fig. 3.

##### Sexual dimorphism and variation.

Females differ from males in the absence of hemipenial swellings at the tail base. The scale counts vary among the type series: scales between supranasals 1–3; supralabials 10–12; infralabials 9 or 10; chin scales 4–7; dorsal scale rows 21–27; ventral scale rows 12–16; precloacal pores absent in females (see Table 2).

**Table 2.** Morphological characters of *Hemiphyllodactylus ziegleri* sp. nov. from Son La Province, Vietnam (measurements in mm, * = regenerated or broken tail, min = minimum, max = maximum, other abbreviations defined in the text).

Characters	HUS.2025.06	HUS.2025.08	IB R.6431	HUS.2025.07	IB R.6433	Min-max	HUS.2025.09	HUS.2025.10	IB R.6432	HUS.2025.11	Min-max
	Holotype	Paratype	Paratype	Paratype	Paratype		Paratype	Paratype	Paratype	Paratype	
**Sex**	M	M	M	M	M		F	F	F	F	
**SVL**	41.9	41.8	41.3	38.7	40	38.7–41.9	49.3	47.2	47	47.5	47–49.3
**TaL**	12.6*	18.6*	32.64	26.5*	26.9*		30.18*	28.7*	31.21	36.0	31.21–36
**TrunkL**	20.08	21.8	20.68	19.5	20.3	19.5–21.8	23.8	23	23.2	24.05	23–24.05
**EyeD**	2.68	2.62	2.56	2.55	2.6	2.55–2.68	3.23	3.11	3.09	2.9	2.9–3.23
**HeadL**	9.62	9.67	9.69	9.32	9.54	9.32–9.69	11.5	11.35	11.26	10.6	10.6–11.5
**HeadW**	7.24	7.08	7.22	6.94	7.02	6.94–7.24	9.1	9	8.9	7.96	7.96–9.1
**Nare-eye length (NarEye)**	3.24	3.2	3.22	3.2	3.29	3.2–3.29	4.09	3.94	3.92	3.5	3.5–4.09
**Snout-eye length (SnEye)**	4.51	4.59	4.58	4.3	4.45	4.3–4.59	5.45	5.44	5.15	5.06	5.06–5.45
**Internarial distance (SnW)**	1.59	1.75	1.77	1.48	1.6	1.48–1.77	2.06	2.12	1.89	1.96	1.89–2.12
**Ear opning diameter (EarD)**	0.91	0.96	0.92	0.9	0.97	0.9–0.97	1.11	1.12	1.08	1.06	1.06–1.12
**EarD/EyeD**	0.34	0.37	0.36	0.35	0.37	0.34–0.37	0.34	0.36	0.35	0.37	0.34–0.37
**Trunk/SVL**	0.48	0.52	0.50	0.50	0.51	0.48–0.52	0.48	0.49	0.49	0.51	0.48–0.51
**HeadL/SVL**	0.23	0.23	0.23	0.24	0.24	0.23–0.24	0.23	0.24	0.24	0.22	0.22–0.24
**HeadW/SVL**	0.17	0.17	0.17	0.18	0.18	0.17–0.18	0.18	0.19	0.19	0.17	0.17–0.19
**HeadW/HeadL**	0.75	0.73	0.75	0.74	0.74	0.73–0.75	0.79	0.79	0.79	0.75	0.75–0.79
**SnEye/HeadL**	0.47	0.47	0.47	0.46	0.47	0.46–0.47	0.47	0.48	0.46	0.48	0.46–0.48
**NarEye/HeadL**	0.34	0.33	0.33	0.34	0.34	0.33–0.34	0.36	0.35	0.35	0.33	0.33–0.36
**EyeD/Head**L	0.28	0.27	0.26	0.27	0.27	0.26–0.28	0.28	0.27	0.27	0.27	0.27–0.28
**SnW/Hea**dL	0.17	0.18	0.18	0.16	0.17	0.16–0.18	0.18	0.19	0.17	0.18	0.17–0.19
**EyeD/NarEye**	0.83	0.82	0.80	0.80	0.79	0.79–0.83	0.79	0.79	0.79	0.83	0.79–0.83
**SnW/HeadW**	0.22	0.25	0.25	0.21	0.23	0.21–0.25	0.23	0.24	0.21	0.25	0.21–0.25
**Dorsal scale row**s	22–23	25–27	23–24	25–26	22–23	22–27	21–22	22–24	22	23–24	21–24
**Ventral scale row**s	12	13	14	13	14	12–14	16	13	13	16	13–16
**Cloacal spurs (CloacS)**	1	1	1	1	1	1–1	1	1	1	1	1–1
**Circumnasal (CircN)**	3	3	3	3	3	3–3	3	3	3	3	3–3
**Scales between supranasals (SnS)**	3	3	3	3	3	3–3	3	2	3	1	1–3
**Supralabials (Sublab)**	10/10	10/10	10/10	11/10	10/10	10–11	12/12	12/11	11/12	11/11	11–12
**Infralabials (Inflab)**	9/9	9/9	9/9	9/9	9/9	9	10/10	9/9	9/10	10/10	9–10
**Chin scale (Chin)**	4	7	7	7	4	4–7	7	7	7	7	7
**Lamella formula**											
**forelimbs (pairs only)**	3-4-4-4	3-4-4-4	3-4-4-4	3-4-4-4	3-4-4-4	3-4-4-4	3-4-4-4	3-4-4-4	3-4-4-4	3-4-4-4	3-4-4-4
**hindlimbs (pairs only)**	4-4-4-4	4-4-4-4	4-5-5-5	4-4-5-4	4-5-5-5-5	4-4/5-4/5-4/5	4-5-5-5	4-5-5-5	4-5-5-5	4-4-5-4	4-4/5-4/5-4/5
**First digit**											
**forelimbs**	5	4	3	4	5	3–5	5	5	5	4	4–5
**hindlimbs**	6	5	5	5	5	5–6	4	5	5	4	4–5
**Precloacal pores**	21	21	21	21	23	21–23	0	0	0	0	0

##### Distribution.

*Hemiphyllodactylus ziegleri* sp. nov. is currently only known from the type locality in Nong Vai Village, Co Ma Commune, Son La Province, Vietnam.

##### Etymology.

We name the new species in honor of Prof. Dr. Thomas Ziegler from Cologne Zoo / Institute of Zoology, University of Cologne, Germany for his outstanding contribution to biodiversity research and conservation in Vietnam. For the common names we suggest Ziegler’s Slender Gecko (English) and Thạch sùng dẹp ziegler (Vietnamese).

##### Natural history.

Specimens were found between 19:30 and 22:00, on limestone cliffs and an electric pole in the corn field, ~ 1.0–2.5 m above the ground, at an elevation of 1020 m a.s.l. The surrounding habitat was disturbed evergreen karst forest of medium hardwood and shrub. The humidity was ~ 80–90% and the air temperature ranged from 22 to 29 °C.

##### Comparisons.

Morphologically, the new species is similar to *Hemiphyllodactylus bonkowskii*, *H. houaphanensis*, *H. kiziriani*, *H. ngocsonensis*, and *H. yunnanensis*. However, *Hemiphyllodactylus ziegleri* is distinguished from *H. bonkowskii* by having a smaller ratio of HeadW/HeadL (0.73–0.79 vs 0.81–0.88 in *H. bonkowskii*), more precloacal and femoral pores in males (21–23 vs 19 in *H. bonkowskii*), and the absence of a cream-colored venter (vs present in *H. bonkowskii*); from *H. houaphanensis* by having a greater ratio of EarD/EyeD (0.34–0.37 vs 0.30–0.33 in *H. houaphanensis*), smaller ratio of HeadL/SVL (0.22–0.24 vs 0.25–0.28 in *H. houaphanensis*), greater ratio of SnEye/HeadL (0.46–0.48 vs 0.37–0.39 in *H. houaphanensis*), smaller ratio of EyeD/NarEye (0.79–0.83 vs 0.89–0.96 in *H. houaphanensis*), fewer precloacal and femoral pores in adult males (21–23 vs 25 in *H. houaphanensis*), fewer chin scales (4–7 vs 8–9 in *H. houaphanensis*), and more circumnasal scales (3 vs 2 in *H. houaphanensis*); from *H. kiziriani* by having a greater ratio of HeadL/SVL (0.22–0.24 vs 0.17–0.18 in *H. kiziriani*), smaller ratio of HeadW/HeadL (0.73–0.79 vs 0.93–1.10 in *H. kiziriani*), fewer circumnasal scales (3 vs 4 in *H. kiziriani*), more precloacal and femoral pores in males (21–23 vs 10–13 in *H. kiziriani*); from *H. ngocsonensis* by having a smaller ratio of HeadW/HeadL (0.73–0.79 vs 0.80–0.87 in *H. ngocsonensis*), more precloacal and femoral pores in males (21–23 vs 20 in *H. ngocsonensis*), and more dorsal scale rows (22–27 vs 19–21 in *H. ngocsonensis*); and from *H. yunnanensis* Boulenger by having more dorsal scale rows (21–27 vs 8–14 in *H. yunnanensis*), more ventral scale rows (12–16 vs 5–7 in *H. yunnanensis*), more precloacal and femoral pores in adult males (21–23 vs 0–20 in *H. yunnanensis*), and greater ratios of SnW/HeadW, EyeD/NarEye, SnW/HeadL, and EyeD/HeadL (0.21–0.25 vs 0.15–0.19, 0.79–0.83 vs 0.64–0.72, 0.16–0.19 vs 0.13–0.15, and 0.27–0.28 vs 0.20–0.24, respectively in *H. yunnanensis*).

*Hemiphyllodactylus ziegleri* sp. nov. differs from *H. banaensis* Ngo, Grismer, Pham & Wood by having fewer scales between supranasals (2–3 vs 4–11 in *H. banaensis*) and more dorsal scale rows (21–27 vs 17–20 in *H. banaensis*); from *H. changningensis* Guo, Zhou, Yan & Li by having more dorsal scale rows (21–27 vs 11–15 in *H. changningensis*), and more ventral scale rows (12–16 vs 6–8 in *H. changningensis*); from *H. dushanensis* Zhou & Liu by having fewer chin scales (4–7 vs 8–10 in *H. dushanensis*), more dorsal scale rows (21–27 vs 14–15 in *H. dushanensis*), more ventral scale rows (12–16 vs 8–9 in *H. dushanensis*), and fewer precloacal and femoral pores in males (21–23 vs 24–26 in *H. dushanensis*); from *H. dupanglingensis* by having more dorsal scale rows (21–27 vs 14–15 in *H. dupanglingensis*), more ventral scale rows (12–16 vs 10–11 in *H. dupanglingensis*), and fewer chin scales (4–7 vs 11 in *H. dupanglingensis*); from *H. hongkongensis* Sung, Lee, Ng, Zhang & Yang by having more dorsal scale rows (21–27 vs 12–15 in *H. hongkongensis*), more ventral scale rows (12–16 vs 9–10 in *H. hongkongensis*), and fewer precloacal and femoral pores in males (21–23 vs 24–25 in *H. hongkongensis*); from *H. huishuiensis* Yan, Lin, Guo, Li & Zhou by having fewer chin scales (4–7 vs 8–10 in *H. huishuiensis*), more dorsal scale rows (21–27 vs 13–15 in *H. huishuiensis*), and more ventral scale rows (12–16 vs 7–9 in *H. huishuiensis*); from *H. indosobrinus* Eliades, Phimmachak, Sivongxay, Siler & Stuart by having fewer supralabials (10–12 vs 15 in *H. indosobrinus*), fewer dorsal scale rows (21–27 vs 30 in *H. indosobrinus*), and digital lamellae formula 3444 (forefoot) (vs 4554 in *H. indosobrinus*); from *H. jinpingensis* Zhou & Liu by having more dorsal scale rows (21–27 vs 11–12 in *H. jinpingensis*) and more ventral scale rows (12–16 vs 5–7 in *H. jinpingensis*); from *H. khpoh* Grismer, Sinovas, Quah, Thi, Chourn, Chhin, Hun, Cobos, Geissler, Ching & Murdoch by having fewer chin scales (4–7 vs 16 in *H. khpoh*), and more dorsal scale rows (21–27 vs 15 in *H. khpoh*); from *H. laowozhenensis* Zhou, Wang, Cui, Zhang, Shen, Li, Liu & Rao by having fewer chin scales (4–7 vs 10–11 in *H. laowozhenensis*), fewer circumnasal scales (2–3 vs 6 in *H. laowozhenensis*), more dorsal scale rows (21–27 vs 15–16 in *H. laowozhenensis*), and more ventral scale rows (12–16 vs 9 in laowozhenensis); from *H. longlingensis* Zhou & Liu by having fewer circumnasal scales (2–3 vs 4–5 in *H. longlingensis*), more dorsal scale rows (21–27 vs 10–14 in *H. longlingensis*), more ventral scale rows (12–16 vs 6–7), and the presence of anteriorly projecting arms on postsacral (vs absent in *H. longlingensis*); from *H. lungcuensis* by having fewer chin scales (4–7 vs 8–10 in *H. lungcuensis*), more dorsal scale rows (21–27 vs 12–17 in *H. lungcuensis*), and more ventral scale rows (12–16 vs 6–11 in *H. lungcuensis*); from *H. nahangensis* by having fewer chin scales (4–7 vs 8–9 in *H. nahangensis*), more subdigital lamellae on the first finger (3–5 vs 3 in *H. nahangensis*), and a higher number of subdigital lamellae on the first toe (4–5 vs 3 in *H. nahangensis*); from *H. serpispecus* Eliades, Phimmachak, Sivongxay, Siler & Stuart by having more ventral scale rows (12–16 vs 10 in *H. serpispecus*), more precloacal and femoral pores in males (21–23 vs 11 in *H. serpispecus*), and fewer cloacal spurs (1 vs 2 in *H. serpispecus*); from *H. typus* Bleeker by having fewer chin scales (4–7 vs 9–14 in *H. typus*) and more dorsal scale rows (21–27 vs 12–19 in *H. typus*); from *H. vanhoensis* by having a greater ratio of HeadW/HeadL (0.73–0.79 vs 0.68–0.72 in *H. vanhoensis*), more dorsal scale rows (22–27 vs 15–19 in *H. vanhoensis*), fewer circumnasal scales (3 vs 5–6 in *H. vanhoensis*), and more precloacal and femoral pores in males (21–23 vs 0 in *H. vanhoensis*); from *H. xiengkhouangensis* by having fewer chin scales (4–7 vs 8–9 in *H. xiengkhouangensis*), more dorsal scale rows (22–27 vs 19–20 in *H. xiengkhouangensis*), and more ventral scale rows (12–16 vs 9–11 in *H. xiengkhouangensis*); from *H. yenchauensis* by having more precloacal and femoral pores in males (21–23 vs 0 in *H. yenchauensis*) and more dorsal scale rows (22–27 vs 21 in *H. yenchauensis*); and from *H. zugi* by having fewer chin scales (4–7 vs 9–12 in *H. zugi*).

## Discussion

During the last five years, 35 additional species have been described within the genus *Hemiphyllodactylus* (Uetz et al. 2025). Eight of these species, i.e., *H. bonkowskii*, *H. dalatensis*, *H. lungcuensis*, *H. nahangensis*, *H. ngocsonensis*, *H. vanhoensis*, *H. cattien*, and *H. yenchauensis* were recently discovered in Vietnam (Do et al. 2020, 2021; Luu et al. 2023, 2024, 2025; Uetz et al. 2025). Our discovery brings the number of *Hemiphyllodactylus* species known from Son La Province to four, and from Vietnam to 12.

Grismer et al. (2013) used the integrative taxonomy to disentangle the complexity of the genus. In particular, the study elevated three subspecies of *H. yunnanensis*, i.e., *H. y. dushanensis*, *H. y. jipingensis*, and *H. y. longlingensis* to the species level based on their morphological and molecular distinctiveness. These authors also suggested that analyzed samples from Yunnan consist of at least five undescribed species. However, due to the lack of specimens and information related to localities of the collected samples, the lineages have not been officially described. Our phylogenetic results show that the new species is supported as a sister species to one of the populations of *Hemiphyllodactylus* from Yunnan, China. It is clear that the region, which comprises southwestern China, northern Lao, and northwestern Vietnam, still harbors a high level of cryptic *Hemiphyllodactylus* diversity (see Agung et al. 2021, 2022).

The discovery of a new species in limestone karsts further highlights the importance of these ecosystems in generating and harboring endemic diversity. Northern Vietnam has one of the most extensive limestone karst systems in Southeast Asian (Clements et al. 2006), it remains insufficiently studied. Recent works, e.g., Nguyen et al. (2017, 2020), Luu et al. (2024, 2025), and Pham et al. (2024), continue to discover new species of *Cyrtodactylus*, *Hemiphyllodactylus*, and *Scincella* from these regions. However, it is likely that additional new species of the genus will be found if more expeditions are conducted in the poorly surveyed sites in the karst system.

The new species is currently known only from Copia Nature Reserve, a protected area established in 2002 in Son La Province. Although the new species has a small range with an estimate of less than 50 km^2^, the area has been experiencing severe habitat degradation primarily as a result of road construction and timber logging. While it is unclear whether these activities significantly threaten its population, they likely will have an adverse effect on the new species. We recommend listing the species as Data Deficient based on the IUCN Red List categories and criteria (IUCN Red List of Threatened Species 2025). Further research is needed to clarify the population status of this species and to determine specific anthropogenic threats at the site.

## Supplementary Material

XML Treatment for Hemiphyllodactylus ziegleri

## Additional information

### Use of AI

No use of AI was reported.

### Author ORCIDs

Anh Van Pham https://orcid.org/0000-0002-6023-3418

Truong Quang Nguyen https://orcid.org/0000-0002-6601-0880

Cuong The Pham https://orcid.org/0000-0001-5158-4526

Hanh Thi Ngo https://orcid.org/0000-0002-5283-6243

Minh Duc Le https://orcid.org/0000-0002-2953-2815

### Data availability

All of the data that support the findings of this study are available in the main text or Supplementary Information.

## Supplementary materials

Supplementary material 1 Specimens used for the phylogenetic analyses and their GenBank accession numbers Data type docx https://binary.pensoft.net/file/1527445This dataset is made available under the Open Database License (http://opendatacommons.org/licenses/odbl/1.0/). The Open Database License (ODbL) is a license agreement intended to allow users to freely share, modify, and use this Dataset while maintaining this same freedom for others, provided that the original source and author(s) are credited. Anh Van Pham, Truong Quang Nguyen, Cuong The Pham, Hanh Thi Ngo, Minh Duc Le

Supplementary material 2 Morphological data used for statistical analyses Data type csv https://binary.pensoft.net/file/1527446This dataset is made available under the Open Database License (http://opendatacommons.org/licenses/odbl/1.0/). The Open Database License (ODbL) is a license agreement intended to allow users to freely share, modify, and use this Dataset while maintaining this same freedom for others, provided that the original source and author(s) are credited. Anh Van Pham, Truong Quang Nguyen, Cuong The Pham, Hanh Thi Ngo, Minh Duc Le

Supplementary material 3 Pair-wise genetic divergence between species included in this study Data type xlsx https://binary.pensoft.net/file/1527447This dataset is made available under the Open Database License (http://opendatacommons.org/licenses/odbl/1.0/). The Open Database License (ODbL) is a license agreement intended to allow users to freely share, modify, and use this Dataset while maintaining this same freedom for others, provided that the original source and author(s) are credited. Anh Van Pham, Truong Quang Nguyen, Cuong The Pham, Hanh Thi Ngo, Minh Duc Le
