# Supplementary material for: Hemiphyllodactylus ziegleri sp. nov. (Squamata, Gekkonidae), a new karst-dwelling gecko species from Son La Province, Vietnam
Source: Zookeys. 2026 Feb 4;1268:75–94. doi: 10.3897/zookeys.1268.174678 (PMC12895188; doi:10.3897/zookeys.1268.174678)
Supplement: Supplementary material 1 — Specimens used for the phylogenetic analyses and their GenBank accession numbers [file zookeys-1268-075_article-174678__-s001.docx]

***Hemiphyllodactylus ziegleri* sp. nov. (Squamata, Gekkonidae), a new karst-dwelling gecko species from Son La Province, Vietnam**

**Table 1.** A list of specimens used for the phylogenetic analyses and their GenBank accession numbers.

| **Species** | **Voucher No./Field No.** | **Location** | **GenBank No.** |
| --- | --- | --- | --- |
| *Hemiphyllodactylus ziegleri* sp. nov. | HUS.2025.07 | Vietnam: Copia NR, Son La | PX725986 |
| *Hemiphyllodactylus ziegleri* sp. nov. | HUS.2025.11 | Vietnam: Copia NR, Son La | PX725985 |
| *Hemiphyllodactylus ziegleri* sp. nov. | HUS.2025.08 | Vietnam: Copia NR, Son La | PX725987 |
| *Hemiphyllodactylus ziegleri* sp. nov. | HUS.2025.06 | Vietnam: Copia NR, Son La | PX725988 |
| *H. banaensis* | ITBCZ 2450 | Vietnam: Ba Na – Nui Chua | KF219783 |
| *H. bonkowskii* | IEBR 4694 | Vietnam: Hang Kia – Pa Co NR, Mai Chau, Hoa Binh | MT415553 |
| *H. bonkowskii* | IEBR 4695 | Vietnam: Hang Kia – Pa Co NR, Mai Chau, Hoa Binh | MT415554 |
| *H. changningensis* | NJNUh00325 | China: Changning, Yunnan | KP732437 |
| *H. changningensis* | NJNUh00326 | China: Changning, Yunnan | KP732438 |
| *H. dupanglingensis* | CSUFT 00401 | China: Dupangling, Hunan | MT576070 |
| *H. dupanglingensis* | CSUFT 00405 | China: Dupangling, Hunan | MT576071 |
| *H. dushanensis* | Isolate N1 | China: Yunnan |  |
| *H. dushanensis* | Isolate N2 | China: Yunnan |  |
| *H. gengmaensis* | 2014002299 | China: Gengma Dai and Wa Autonomous County, Yunnan | PP540022 |
| *H. gengmaensis* | 2014002297 | China: Gengma Dai and Wa Autonomous County, Yunnan | PP540023 |
| *H. harterti* | LSUHC10383 | Malaysia: Bukit Larut | KF219760 |
| *H. harterti* | LSUHC10384 | Malaysia: Bukit Larut | KF219761 |
| *H. hongkongensis* | SYS r001728 | Hong Kong: Aberdeen Country Park | MF893330 |
| *H. hongkongensis* | SYS r001735 | Hong Kong: Aberdeen Country Park | MF893333 |
| *H. houaphanensis* | VNUF R.2020.16 | Laos: Sa Kok, Hiem, Houaphan | PQ008303 |
| *H. houaphanensis* | VNUF R.2020.17 | Laos: Sa Kok, Hiem, Houaphan | PQ008304 |
| *H. huishuiensis* | NJNUh00851 | China: Huishui, Guizhou | KU519707 |
| *H. huishuiensis* | NJNUh00857 | China: Huishui, Guizhou | KU519710 |
| *H. jinhongensis* | KIZ2023579 | China: Mengsong village, Jinghong City | PQ477984 |
| *H. jinhongensis* | KIZ2023605 | China: Mengsong village, Jinghong City | PQ477989 |
| *H. khpoh* | LSUHC 15238 | Cambodia: Phnom Khoph, Banan District, Battambang | PQ046265 |
| *H. kiziriani* | IEBR A.2014.3 | Laos: Luang Prabang | KJ676800 |
| *H. kiziriani* | IEBR A.2014.4 | Laos: Luang Prabang | KJ676801 |
| *H. laowozhenensis* | LW2024R005 | China: Laowo Town, Lushui City, Yunnan | PQ819179 |
| *H. laowozhenensis* | LW2024R006 | China: Laowo Town, Lushui City, Yunnan | PQ819180 |
| *H. lingshuiensis* | SYS r002845 | China: Mt. Diaoluo, Lingshui, Hainan | PV296340 |
| *H. longlingensis* | KIZ2023703 | China: Xinzhai village, Tengchong City | PQ477980 |
| *H. longlingensis* | KIZ2023704 | China: Xinzhai village, Tengchong City | PQ477981 |
| *H. lungcuensis* | IEBR R.5151 | Vietnam: Lung Cu, Dong Van, Ha Giang | OR067853 |
| *H. lungcuensis* | VNUF R.2023.01 | Vietnam: Lung Cu, Dong Van, Ha Giang | OR067854 |
| *H. nahangensis* | IEBR 4741 | Vietnam: Trung Phin, Sinh Long, Na Hang, Tuyen Quang | MT711191 |
| *H. nahangensis* | IEBR 4742 | Vietnam: Trung Phin, Sinh Long, Na Hang, Tuyen Quang | MT711192 |
| *H. ngocsonensis* | IEBR 4689 | Vietnam: Ngoc Son – Ngo Luong NR, Lac Son, Hoa Binh | MT415551 |
| *H. ngocsonensis* | IEBR 4690 | Vietnam: Ngoc Son – Ngo Luong NR, Lac Son, Hoa Binh | MT415552 |
| *H. pardalis* | ZMMU R–16390 | Thailand: Suang Phueng, Ratchaburi | MT656373 |
| *H. pardalis* | Isolate SP-122-1 | Thailand: Suang Phueng, Ratchaburi | MT656374 |
| *H. serpispecus* | NUOL 00476 | Laos: Tham Ngou Leium Cave, Viengxay, Houaphan | MK307996 |
| *H. typus* | MVZ 226500 | Vietnam: Vinh Phuc | KF219798 |
| *H. vanhoensis* | VNUF R.2022.07 | Vietnam: Tan Xuan, Van Ho, Son La | PQ008305 |
| *H. vanhoensis* | VNUF R.2020.09 | Vietnam: Tan Xuan, Van Ho, Son La | PQ008306 |
| *H. xiengkhouangens* | NUOL R.2025.01 | Laos: Xiengkhouang | PV849544 |
| *H. xiengkhouangens* | VNUF R.2025.02 | Laos: Xiengkhouang | PV849545 |
| *H. yanshanensis* | KIZ062102 | China: Yunnan | ON676161 |
| *H. yanshanensis* | KIZ062093 | China: Yunnan | ON676164 |
| *H. yenchauensis* | VNUF R.2025.05 | Vietnam: Chieng Tuong, Yen Chau | PV849542 |
| *H. yenchauensis* | VNUF R.2025.06 | Vietnam: Chieng Tuong, Yen Chau | PV849543 |
| *H. yunnanensis* | Isolate N10 | China: Yunnan | FJ971016 |
| *H. yunnanensis* | Isolate N11 | China: Yunnan | FJ971017 |
| *H. yunnanensis* | Isolate N29 | China: Yunnan |  |
| *H. zalonicus* | ZMMU R 16635 | Myanamar: Zalon Taung National Forest, Ban Mauk, Sagaing | MW039150 |
| *H. zayuensis* | KIZ045786 | China: Jing Che | MW111434 |
| *H. zayuensis* | KIZYPX1520 | China: Jing Che | MW111435 |
| *H. zugi* | IEBR A.2013.21 | Vietnam: Ha Lang, Cao Bang | KF575152 |
| *H. zugi* | ZFMK 94782 | Vietnam: Ha Lang, Cao Bang | KF575153 |
| *H. zhutangxiangensis* | KIZ061157 | China: Zhutangxiang town, Lancang Lahu, Yunnan | MW929173 |
| *H. zhutangxiangensis* | KIZ061158 | China: Zhutangxiang town, Lancang Lahu, Yunnan | MW929174 |
